# Catalpol ameliorates depressive-like behaviors in CUMS mice via oxidative stress-mediated NLRP3 inflammasome and neuroinflammation

**DOI:** 10.1038/s41398-021-01468-7

**Published:** 2021-06-08

**Authors:** Ya-lin Wang, Hao-ran Wu, Shan-shan Zhang, Hong-lei Xiao, Jin Yu, Yuan-yuan Ma, Yao-dong Zhang, Qiong Liu

**Affiliations:** 1 Department of Integrative Medicine and Neurobiology, School of Basic Medical Sciences, Institutes of Brain Science, State Key Laboratory of Medical Neurobiology and Collaborative Innovation Center for Brain Science, Shanghai Medical College, Fudan University, Shanghai, 200032 China; 2 Department of Anatomy, Histology and Embryology, School of Basic Medical Sciences, Shanghai Medical College, Fudan University, Shanghai, 200032 China; 3 State Key Laboratory of Medical Neurobiology, Fudan University, Shanghai, China; 4 Children’s Hospital Affiliated to Zhengzhou University, Henan Neural Development Engineering Research Center, Henan, China; 5 Key Laboratory of Medical Imaging Computing and Computer Assisted Intervention of Shanghai, Shanghai, 200032 China

**Keywords:** Molecular neuroscience, Neuroscience

## Abstract

The purpose of the present study was to investigate whether catalpol exhibited neuroprotective effects in chronic unpredictable mild stress (CUMS) mice through oxidative stress-mediated nucleotide-binding oligomerization domain, leucine-rich repeat, and pyrin-domain-containing 3 (NLRP3) inflammasome and neuroinflammation. Deficits in behavioral tests, including open field test (OFT), forced swim test (FST), and elevated plus-maze test (EPM), were ameliorated following catalpol administration. To study the potential mechanism, western blots, quantitative real-time PCR (qRT-PCR) analysis and immunofluorescence imaging were performed on the hippocampus samples. We found that the defects of behavioral tests induced by CUMS could be reversed by the absence of NLRP3 and NLRP3 inflammasome might be involved in the antidepressant effects of catalpol on CUMS mice. Similar to the NLRP3 inflammasome, the expression of interleukin-1 beta (IL-1β), tumor necrosis factor alpha (TNF-α), and inducible nitride oxide synthase (iNOS) were increased after CUMS. The current study demonstrated that catalpol possessed anti-inflammatory effect on CUMS mice and inhibited microglial polarization to the M1 phenotype. In addition, the activity of mitochondrial oxidative stress might be involved in the NLRP3 activation, which was proved by the downregulation of NLRP3, apoptosis-associated speck-like protein containing a CARD (ASC), and cleaved IL-1β, after the administration of mitochondrion-targeted antioxidant peptide SS31. Taken together, we provided evidence that catalpol exhibited antidepressive effects on CUMS mice possibly via the oxidative stress-mediated regulation of NLRP3 and neuroinflammation.

## Introduction

Depression is a common disease which endangers both the physical and psychological health of people worldwide, contributing to a heavy social burden and severe suicide tendency^1^. Serotonin selective reuptake inhibitors (SSRIs) are currently the most frequently prescribed therapeutic agents in the treatment of depression. However, long-term use of SSRIs has brought out side effects over time^2,3^. Furthermore, 30–40% of depressive patients do not respond to the therapy and that the incidence of depression is constantly growing, the search for safer and more effective antidepressant therapies is becoming an urgent need^4^.

Catalpol, an iridoid glucoside, is widely distributed in many plant families and is primarily obtained from the root of *Rehmannia glutinosa* *Libosch*, which is commonly used in traditional Chinese medicine^5^. Catalpol has been shown to exert a wide variety of pharmacological effects, such as anti-inflammatory, antitumor, and antioxidant activities^6,7^. Previous studies have displayed that catalpol can alleviate depressive-like behavior, dampen inflammatory activity, and inhibit the overproduction of reactive oxygen species (ROS)^8–10^. Growing evidence indicates that catalpol may play an antidepressant role through its anti-inflammatory and antioxidative effect^8,11^. However, the biological mechanism of depression remains unclear. Therefore, the present study was aimed to investigate the potential mechanism by which catalpol might relieve depressive symptoms.

Depression has been widely considered as a neuroinflammation disease in recent years. Stress increases proinflammatory cytokines in the peripheral and central nervous system (CNS), as well as the activation of microglia in the brain, characterized by the increase of its cell surface marker ionized calcium binding adapter molecule-1 (Iba-1), thereby elevating sensitivity to immunostimulation^12,13^. Cytokines, such as interleukin-1 beta (IL-1β) and tumor necrosis factor alpha (TNF-α), are important in cell signaling as inflammatory mediators and they also affect neurotransmitter systems, brain functionality, and mood^14–16^. Several studies have displayed that proinflammatory cytokines increased in major depressive disorders (MDDs)^17–20^. Furthermore, anti-inflammatory agents, such nonsteroidal anti-inflammatory drugs, have shown antidepressant treatment effects^21–23^. A recent study has reported that catalpol can ameliorate chronic unpredictable mild stress (CUMS)-induced depressive-like behavior, and suggests that its mechanisms may partially be ascribed to downregulating cyclooxygenase-2 expression^8^. However, few studies have been undertaken to investigate the mechanism by which catalpol mediates neuroinflammation in depression.

As the most important cytokine, IL-1β plays a key role in the development of neuroinflammation and depression^24^. Previous studies have reported that IL-1β knock-down significantly attenuated the memory deficits and anxiety- and depressive-like behaviors in mice^25,26^. NLRP3 inflammasome and its cleaved caspase-1 are critical for IL-1β maturation. After assembly, the NLRP3 complex is responsible for the proteolytic cleavage of pro-IL-1β into the active and secreted forms IL-1β, driving proinflammatory responses that culminate in cellular damage, autophagy, and pyroptosis^27^. Recent studies have found that mitochondria may be relative to activation of the NLRP3 inflammasome. Moreover, current studies have reported that ROS derived from mitochondria is a key signal for regulating NLRP3 inflammasome activation^28^.

Based on these observations above, we hypothesized that catalpol may exhibit antidepressive effects on CUMS mice through the oxidative stress-mediated regulation of NLRP3 inflammasome and neuroinflammation. We demonstrate that catalpol indeed attenuates depressive-like behaviors in CUMS mice. NLRP3 inflammasome activation, proinflammatory cytokines, and ROS released after CUMS were all dampened following the administration of catalpol. In addition, administration of mitochondrion-targeted antioxidant peptide SS31 ameliorated CUMS-induced depressive-like behavior possibly via downregulating NLRP3 inflammasome activation and neuroinflammation. To our knowledge, this is the first report showing that catalpol, which is widely distributed in the root of *Rehmannia glutinosa Libosch*, downregulating oxidative stress-mediated NLRP3 inflammasome activation and neuroinflammation, plays an antidepressant role in CUMS mice.

## Materials and methods

### Animals

C57BL/6 male mice weighing 19–22 g were obtained from Shanghai SLAC Laboratory Animal Corp. Ltd. C57BL/6 NLRP3 KO mice were generated by Shanghai Model Organisms Center, Inc. Mice were housed in standard cages (five animals in each cage) for 1 week under standard conditions of constant temperature (~23 °C) and a 12 h light/dark cycle, with free access to food and water before the experiment. Animals that developed illnesses during the experiments were excluded from the study. All the following behavioral tests were performed by the same experimenter blinded to the group assignment to minimize the differences between experimenters.

This study was carried out in accordance with the National Institutes of Health Guide for the Care and Use of Laboratory Animals. The protocol was approved by the Animal Ethics Committee of Shanghai Medical College, Fudan University, Shanghai, China (20120302-107).

### Drugs and administration

Catalpol (Beyotime Biotechnology Co., Ltd) and mitochondrion-targeted antioxidant peptide SS31 (ChinaPeptides Co., Ltd) were dissolved in saline before administration (Catalpol: 20 mg/kg, SS31: 5 mg/kg). The doses were optimized based on prior assessments of the best dose–response relationship in mice. The behavioral tests and western blot data showed no statistical differences in comparison with control mice administered either with saline.

### Animal treatment

Briefly, mice were singly housed during the experimental procedures with two random stressors per day. The mild stressors included: the cage being tilted at 45° for 24 h, 24 h food deprivation, 24 h water deprivation, wet bedding for 24 h, swimming in 4 °C water for 5 min, shaking at 120 rpm for 30 min, restraint in a 50 ml tube for 6 h, and illumination throughout the night (overnight illumination occurred twice per week). Control mice were group-housed under normal conditions. After 5 weeks of CUMS, two CUMS groups (each *n* = 8) were administered with catalpol or vehicle once daily for 4 weeks. One CUMS group was treated with vehicle (saline) in order to mimic manipulations. Treatment of SS31 was also carried out with simultaneous 5-week CUMS procedure (5 mg/kg. ip. qd). After the final day of administration, behavioral tests were performed.

### Behavioral tests

#### Forced swimming test (FST)

The mice were taken from their home cage and placed individually in a glass cylinder (25 cm high, 10 cm in diameter) filled with 19 cm water at 24 ± 1 °C. During the test, mice were placed into the water for 6 min, and the total duration of immobility was recorded for the last 4 min. The duration of immobility was defined as floating or only making slight movements to keep the head above the water.

#### Open field test (OFT)

The OFT is used to measure physical condition and anxiety-like behavior in a brightly lit open area^29^. The OFT was performed in dim light (15 lux). The open box apparatus was 50 cm × 50 cm × 40 cm. Mice were gently placed into the center of the arena and allowed to explore for 5 min. The digitized image of the movements of each mouse was recorded using a camera. The activity behaviors were tracked by a video and analyzed by software (Shanghai Mobile Datum Information Technology Company, Shanghai, China). The experimental apparatus was cleaned between consecutive tests.

#### Elevated plus-maze test (EPM)

The EPM test consists of four arms (5 × 30 cm). Two closed arms have 20-cm-high walls and the other arms are left open (open arm). The maze was elevated 40 cm above the floor. Mice were placed in the center of the maze facing an open arm, and allowed free access to four arms for 5 min. Open-arm time ([time in open arms]/[time in total arms] × 100) was calculated. The maze was rinsed between sessions with 75% alcohol and dried with a towel.

### Western blot analysis

The hippocampal tissue was homogenized in radioimmunoprecipitation assay lysis buffer (Thermo Scientific) and lysed for 30 min. The supernatant was centrifuged at 12,000 rotations per minute (rpm) for 20 min. Then, the total protein concentration of the supernatants was assessed using a BCA kit (Pierce Scientific). The protein in each sample was separated by electrophoresis and transferred to polyvinylidene fluoride membranes. After blocking the membranes with 5% milk for 2 h, they were incubated overnight with antibodies against, NLRP3 (1:1000, Abcam), cleaved caspase-1 (1:1000, Abcam), ASC (1:200, Santa Cruz), cleaved IL-1β (1:1000, R&D System), and β-actin (1:20,000, Cell Signaling). Secondary antibodies (1:10,000, EarthOx) were incubated for 2 h at room temperature. The signal was captured on an ImageQuant LAS4000 mini image analyzer (GE Healthcare, Buckinghamshire, UK), and the band levels were quantified using ImageJ software (NIH, Bethesda, MD, USA). To control sampling errors, the ratio of band intensities to β-actin was obtained to quantify the relative protein expression level.

### Quantitative real-time PCR (qRT-PCR)

Total RNA was extracted using TRIzol Reagent (Invitrogen). cDNA was synthesized with a PrimeScript™ RT Master Mix Kit (Takara Bio, Tokyo, Japan). qRT-PCR was performed on a 7500 Fast Real-Time PCR System (AB Applied Biosystems, USA) using SYBR Green Realtime PCR Master Mix (Takara Bio, Tokyo, Japan). The specificity of amplification was assessed for each sample by melting curve analysis. Relative quantification was performed using standard curve analysis. The quantification data are presented as a ratio to the control level. The following primers were used: IL-1β, 5′-GTA CAA GGA GAA CCA AGC AA-3′ (sense) and 5′-CCG TCT TTC ATT ACA CAG GA-3′ (antisense); IL-6, 5′-CCA ATG CTC TCC TAA CAG AT-3′ (sense) and 5′-TGT CCA CAA ACT GAT ATG CT-3′ (antisense); IL-10, 5′-GGG AAG AGA AAC CAG GGA GA-3′ (sense) and 5′-GGG GAT GAC AGT AGG GGA AC-3′ (antisense); CD206, 5′-GTG CCT ACT GCC TGC CCT AA-3′ (sense) and 5′-TCC CAT CGC TCC ACT CAA AG-3′ (antisense); inducible nitride oxide synthase (iNOS), 5′-GCA CAG AGG GCT CAA AGG-3′ (sense) and 5′-CAC ATC GCC ACA AAC ATA AA-3′ (antisense); and GAPDH, 5′-ATG ACC CCT TCA TTG ACC-3′ (sense) and 5′-GAA GAT GGT GAT GGG ATT TC-3′ (antisense).

### Immunofluorescence imaging

To prepare brain slices, mice were anesthetized (4 ml/kg, 10%chloral hydrate) and perfused intracardially with 0.9% saline followed by 4% paraformaldehyde (PFA) in PBS. The brains were removed and postfixed in 4% PFA at 4 °C overnight, immersed in 20% sucrose (4% PFA as solvent) followed by 30% sucrose (in 0.1 M PBS), cut into 40-μm-thick sections (CM1850; Leica Microsystems, Wetzlar, Germany), and then incubated with the following primary Ab: anti-Iba-1 antibody (1:500). The sections were then incubated with the appropriate Alexa Fluor-conjugated secondary Abs, and then counterstained with DAPI to visualize the cell nuclei. Immunofluorescence sections were observed with a Leica SP5 fluorescence microscope and photographed with a CCD spot camera for data analysis. The same region per hippocampal section and three sections per animal were counted, using ImageJ, in a blinded fashion. Cells were counted as Iba-1/DAPI double-positive if two signals were colocalized.

### Measurement of ROS generation

Intracellular ROS level was assayed by the fluorescent probe dichlorodihydrofluorescein diacetate (DCFH-DA) (Beyotime, China). After the introduction of different stimulus, cells were incubated with 50 μM DCFH-DA at 37 °C for 20 min in the dark. Then, the cells were washed twice using cold PBS. The average fluorescence intensity was analyzed by using an image analysis system (ImageJ, National Institutes of Health).

### Statistical analysis

All data are presented as means ± SD. Group differences were analyzed using a one-way analysis of variance with a Bonferroni correction to adjust for multiple testing. *p* < 0.05 was considered statistically significant. Statistical analysis was carried out using GraphPad Prism 6.0 software.

## Results

### Catalpol significantly ameliorated depressive-like behaviors in CUMS mice

**Fig. 1:**
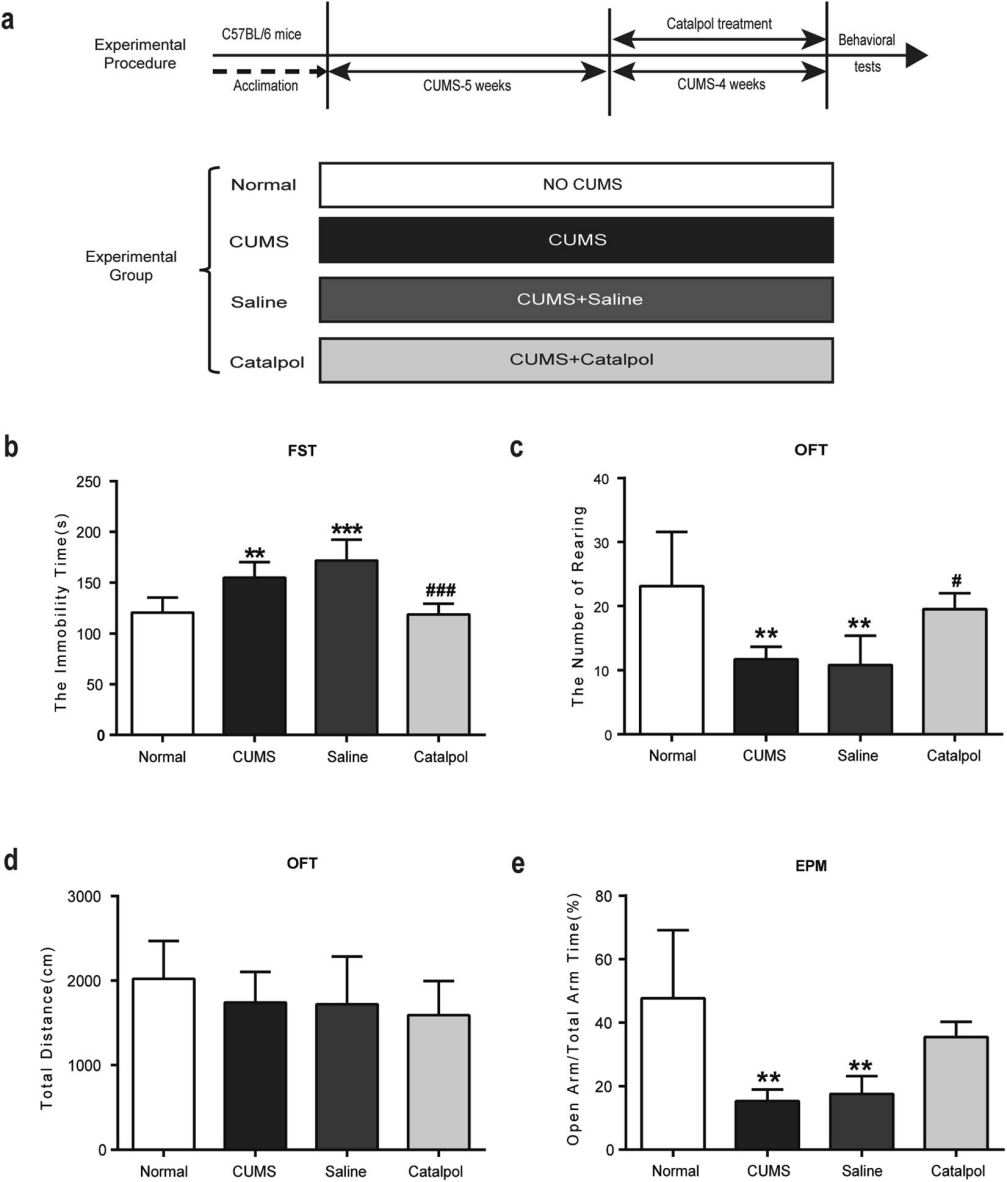
Catalpol conferred antidepressive effects on behavioral tests. **a** The experimental paradigm and the experimental group. **b** The time spent immobile in the FST (*n* = 8/group). **c** Rearing numbers in the OFT (*n* = 8/group). **d** Total distance traveled in the OFT (*n* = 8/group). **e** Open-arm time proportion in the EPM test (*n* = 8/group). All data are expressed as the mean ± SD. **p < 0.01, ***p < 0.001, compared with control group; ^#^*p* < 0.05, ^###^*p* < 0.001, compared with vehicle group.

Four independent groups of animals were used to evaluate the antidepressant effects of catalpol on CUMS mice. The timeline is shown in Fig. 1a. The FST is extensively used for the evaluation of antidepressant activity^30^. As expected, CUMS remarkably increased the immobility time in FST (Fig. 1b) and decreased the number of rearing in OFT (Fig. 1c). There was no difference among groups for the total distance detection in OFT (Fig. 1d). A significant reduction in the portion of time spent in the open arms and total arm time after CUMS procedure was observed (Fig. 1e). After catalpol administration, CUMS-induced increase in the immobility time in FST was significantly reversed (Fig. 1b). In OFT, catalpol treatment significantly increased the number of rearing (Fig. 1c). Figure 1d shows that the total distance for each group was not significantly different after catalpol administration. In EPM, the portion of time spent in the open arms and total arm time was improved after the administration of catalpol than in CUMS or saline group but with no significance (Fig. 1e). The alleviations of these behavioral tests indicated the antidepressive effects of catalpol in CUMS mice.

### Catalpol attenuated NLRP3 inflammasome activation in the hippocampus of CUMS mice

Neuroinflammatory process activated in MDD. The NLRP3 inflammasome, an inflammatory signaling molecular complex, plays a crucial role in depression^31^. Therefore, effects of catalpol on neuroinflammation were investigated in CUMS mice.

**Fig. 2:**
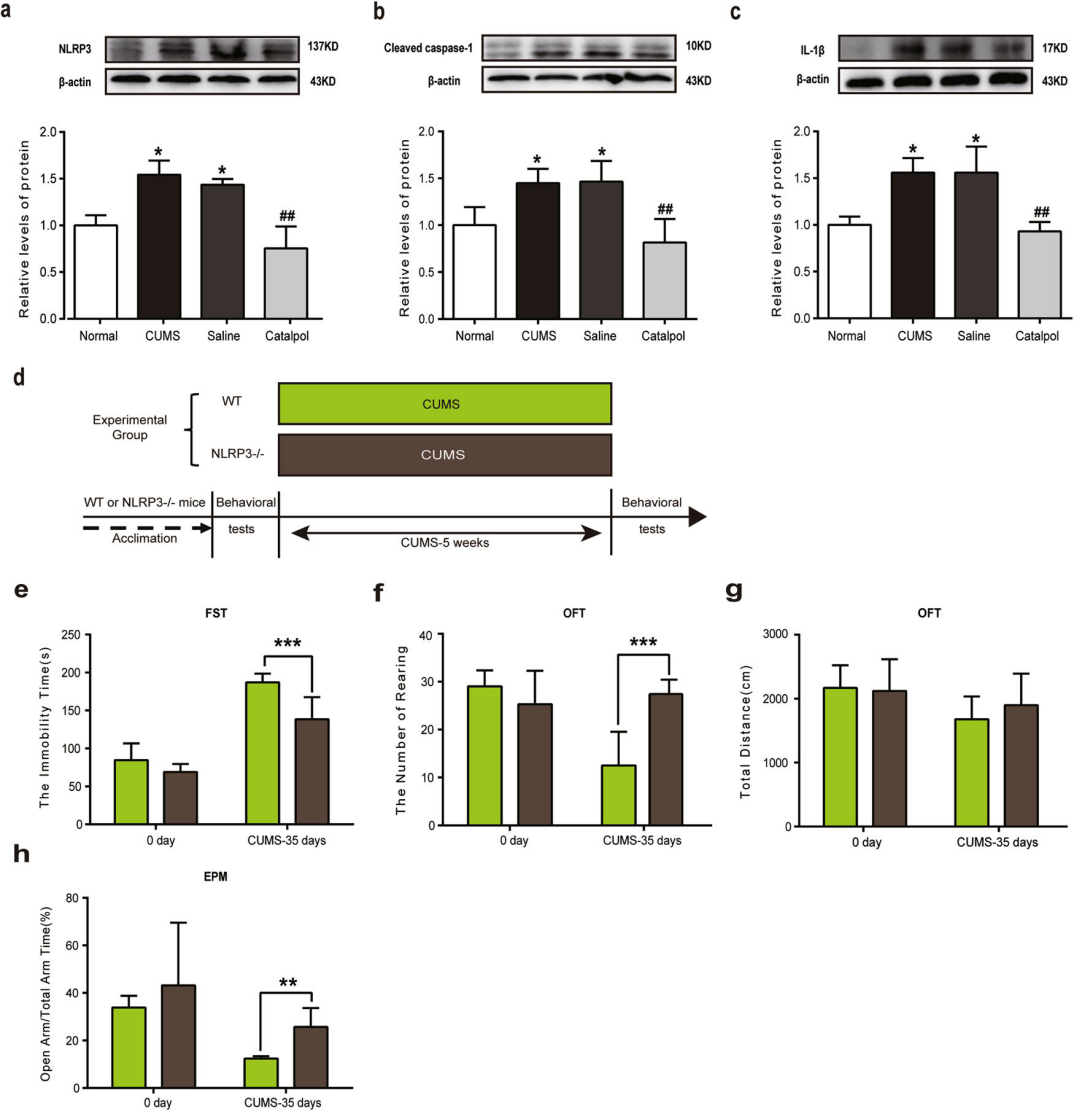
The NLRP3 inflammasome might be involved in antidepressive effect of catalpol. **a–c** Detection of NLRP3 inflammasome in the hippocampus of CUMS mice. Western blot analysis of (**a**) NLRP3 (*n* = 6–8/group), (**b**) cleaved caspase-1 (*n* = 6–8/group), and (**c**) Il-1β (*n* = 6–8/group). All data are expressed as the mean ± SD. *p < 0.05, compared with control group; ^##^*p* < 0.01, compared with vehicle group. **d**–**h** In order to detect whether or not NLRP3 inflammasome plays a key role in CUMS-induced depression, NLRP3 KO mice were used in this experiment. **d** The experimental paradigm and the experimental group. **e** The time spent immobile in the FST (*n* = 10/group). **f** Rearing numbers in the OFT (*n* = 10/group). **g** Total distance traveled in the OFT (*n* = 10/group). **h** Open-arm time proportion in the EPM test (*n* = 10/group). All data are expressed as the mean ± SD. **p < 0.01, ***p < 0.001, compared with wild-type group.

The expression of NLRP3, cleaved caspase-1, and IL-1β were examined to further study the underlying mechanism of the anti-inflammatory effects of catalpol in CUMS mice. As shown in Fig. 2a–c, there’s a significant increase of NLRP3, cleaved caspase-1, and IL-1β in the hippocampus of CUMS mice. After the administration of catalpol, the levels of NLRP3, cleaved caspase-1, and IL-1β in the hippocampal region of mice were remarkably downregulated (Fig. 2a–c). Western blot analysis showed that the NLRP3 inflammasome pathway was activated due to the increasing expression of NLRP3 inflammasome complex.

To further study the role of NLRP3 in depression, NLRP3 KO mice were used for behavioral tests (animal treatment paradigm exhibited in Fig. 2d). Wild-type and *Nlrp3−/−* mice were all treated with chronic stress for 5 weeks. The behavior tests were performed before and after the CUMS. The results showed that there was no significant difference between the two groups before chronic stress (Fig. 2e–h), but the depressive- and anxiety-like behaviors of wild-type CUMS mice were significantly more serious than that of *Nlrp3−/−* CUMS mice, suggesting less immobile time in FST and more rearings in OFT (Fig. 2e, f). There was no difference between groups of total distance in OFT (Fig. 2g). A significantly higher portion of time spent in the open arms and total arm time was observed in *Nlrp3−/−* CUMS mice than in wild-type CUMS mice (Fig. 2h). It is indicated that *Nlrp3−/−* could partly inhibit the depressive- and anxiety-like behaviors induced by CUMS. These results confirmed that catalpol may exert antidepressant-like effect via NLRP3 inflammasome pathway.

### Catalpol dampened neuroinflammation and mitochondrial oxidative stress in the hippocampus of CUMS mice

**Fig. 3:**
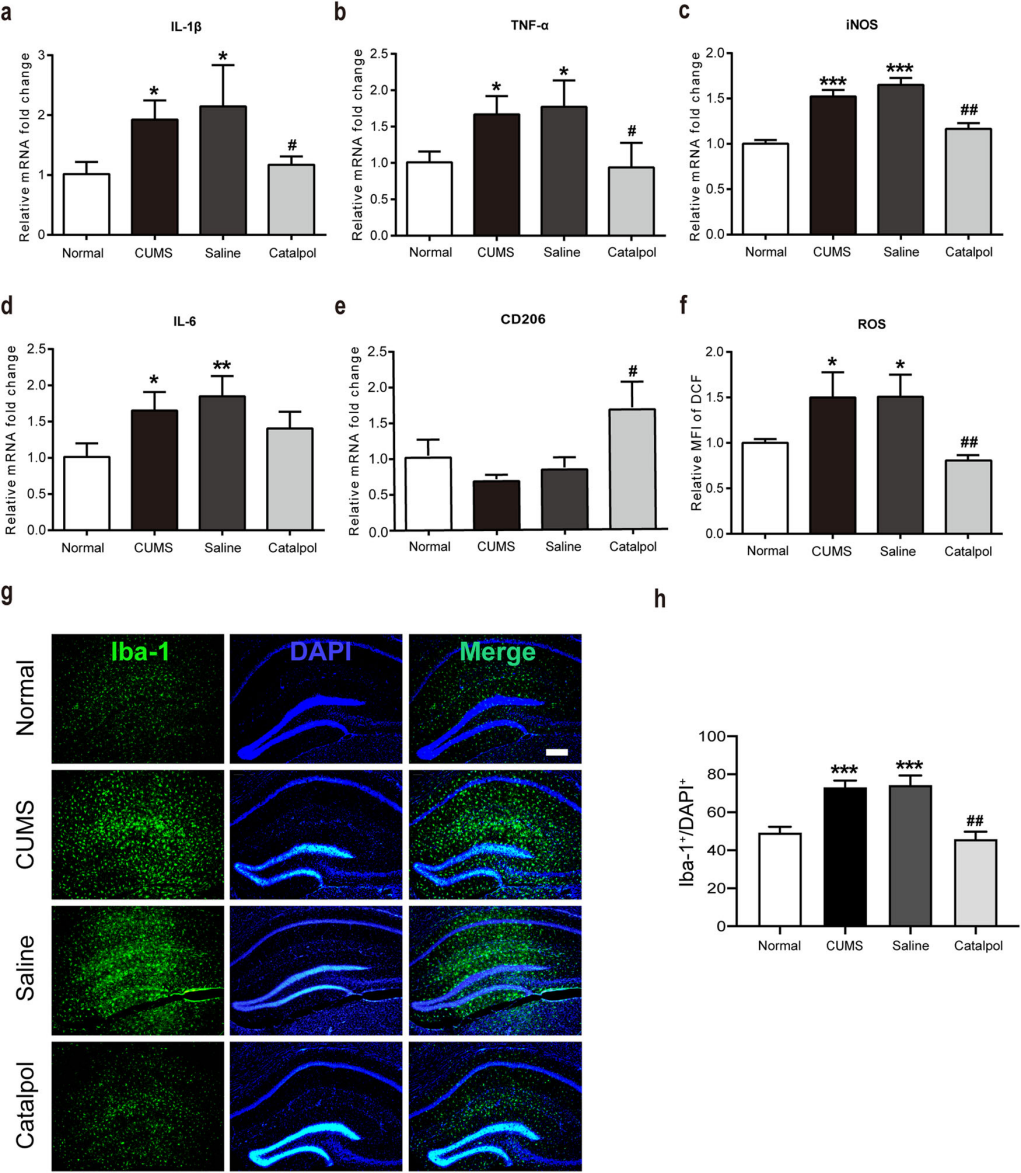
Catalpol dampened neuroinflammation and mitochondrial oxidative stress in the hippocampus of CUMS mice. **a**–**e** The expression of cytokines were assessed by qRT-PCR, the level of IL-1β, TNF-α, iNOS, IL-6 was downregulated significantly by catalpol, and the level of CD206 was also detected. (*n* = 5–8/group). **f** To detect the mitochondrial oxidative stress level, the average fluorescence intensity of DCF was analyzed (*n* = 5–8/group). **g** Immunofluorescence staining of hippocampal sections. Iba-1 (green), DAPI (blue); scale bar, 100 μm. **h** Qualification of Iba-1 immunofluorescence density (*n* = 5–8/group). All data are expressed as the mean ± SD. *p < 0.05, **p < 0.01, ***p < 0.001, compared with control group; ^#^*p* < 0.05, ^##^*p* < 0.01, compared with vehicle group.

Meanwhile, concentrations of proinflammatory cytokine proteins were measured by qRT-PCR. As depicted in Fig. 3, proinflammatory cytokines were significantly increased by CUMS (Fig. 3a–d). The overexpression of IL-1β and TNF-α were all remarkably reversed by the administration of catalpol (Fig. 3a, b).

Catalpol treatment significantly downregulated the level of iNOS, which is not only a proinflammatory cytokine but also a marker of proinflammatory microglial (Fig. 3c). However, the administration of catalpol had no significant effect on the production of IL-6 (Fig. 3d). The marker of anti-inflammatory microglial was also evaluated. As illustrated in Fig. 3e, the evaluated level of CD206 was dramatically increased in the hippocampus of calpol-treated CUMS mice, indicating an upregulating effect on anti-inflammatory microglial by catalpol.

The level of ROS was measured to evaluate the mitochondrial oxidative stress in the hippocampus. A high oxidative stress level was detected by significantly increased expression of ROS after CUMS. Catalpol treatment significantly reduced the ROS accumulation induced by CUMS which indicated that catalpol might exert an effect against oxidative stress (Fig. 3f).

To further clarify neuroinflammation in CUMS mice, we evaluated the expression of the microglial cell surface marker Iba‐1 in the hippocampus by immunofluorescence. There were significantly more Iba‐1‐labeled microglial cells in the hippocampus of CUMS mice than in control mice (Fig. 3g, h), and most of the microglia showed ameboid features, characterized by enlarged cell bodies and synaptic retraction (Fig. 3g). The CUMS-induced microglia activation was significantly renormalized after the administration of catalpol (Fig. 3g, h). Briefly, these results suggest that catalpol played a role in dampening neuroinflammation and mitochondrial oxidative stress in the hippocampus of CUMS mice.

### Inhibition of mitochondrial oxidative stress ameliorated CUMS-induced depressive-like behavior

**Fig. 4:**
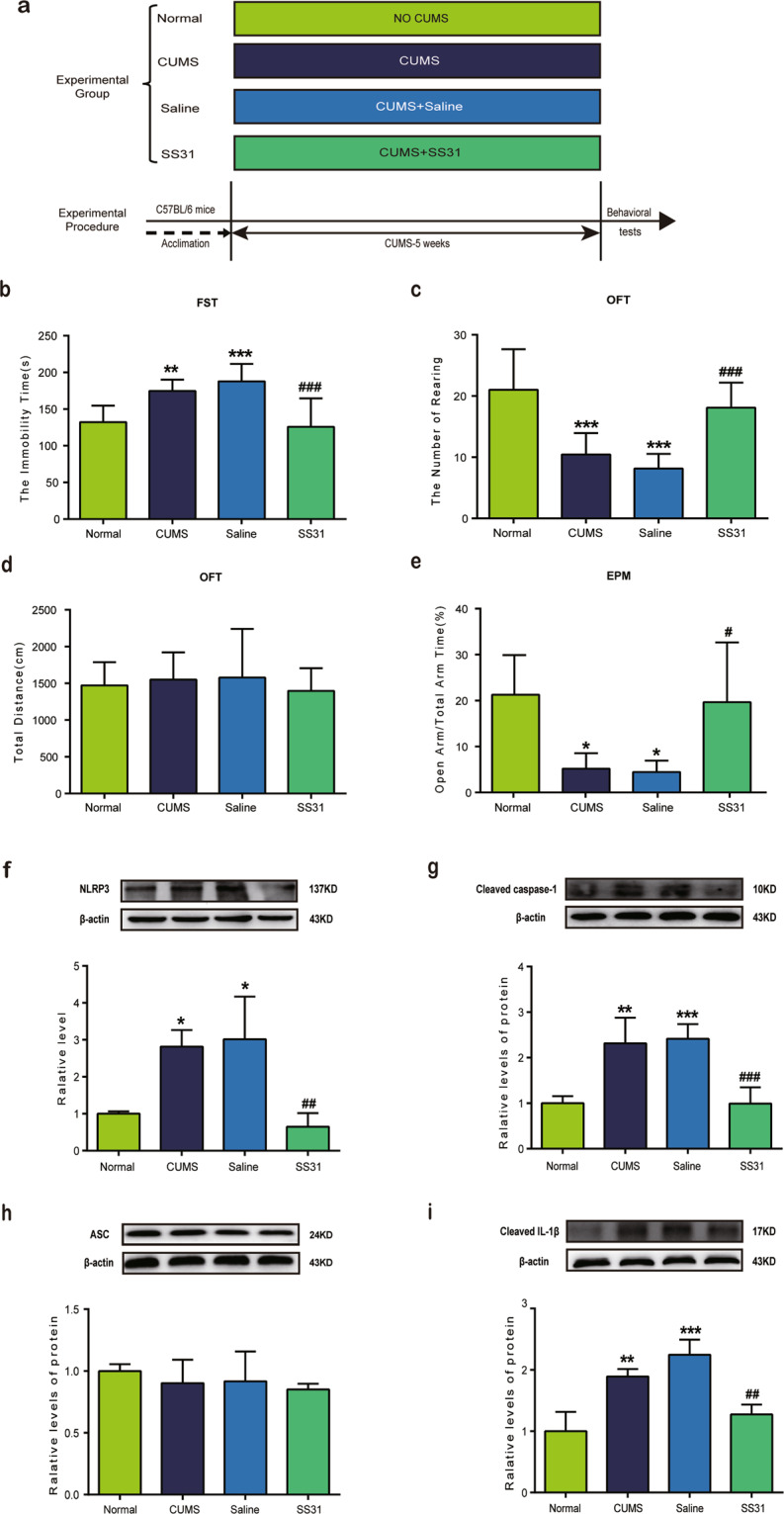
Mitochondrial oxidative stress might be involved in the effects of catalpol on CUMS-induced depressive-like behavior and NLRP3 activation. **a** The experimental paradigm and the experimental group. **b** The time spent immobile in the FST (*n* = 10/group). **c** Rearing numbers in the OFT (*n* = 10/group). **d** Total distance traveled in the OFT (*n* = 10/group). **e** Open-arm time proportion in the EPM test (*n* = 10/group). **f–i** Detection of NLRP3 inflammasome in the hippocampus of CUMS mice. Western blot analysis of (**f**) NLRP3 (*n* = 6–8/group), (**g**) cleaved caspase-1 (*n* = 6–8/group), (**h**) ASC (*n* = 6–8/group), and (**i**) cleaved IL-1β (*n* = 6–8/group). All data are expressed as the mean ± SD. *p < 0.05, **p < 0.01, ***p < 0.001, compared with control group; ^##^*p* < 0.01, ^###^*p* < 0.001, compared with vehicle group.

Here, SS31 was used to assess the role of mitochondrial oxidative stress in CUMS-induced depressive-like behavior (animal treatment paradigm exhibited in Fig. 4a). After a 5-week CUMS procedure, CUMS significantly increased the immobility time in FST (Fig. 4b) and decreased the number of rearing in OFT (Fig. 4c). In OFT, there is no significant difference between groups on the total distance moved (Fig. 4d). A remarkable reduction in the portion of time spent in the open arms and total arm time after CUMS procedure was also observed (Fig. 4e). Treatment with SS31 significantly renormalized the behavioral deficits of CUMS mice, indicated by spending less immobile time and more time struggling in FST (Fig. 4b). Meanwhile, SS31 treatment significantly increased the rearing times in OFT (Fig. 4c). Figure 4d shows that the total distance for each group was not significantly different after SS31 administration. In EPM, the portion of time spent in the open arms and total arm time were also remarkably increased after the administration of catalpol in CUMS mice (Fig. 4e). These results indicated that catalpol might ameliorate depressive-like behaviors via the regulation of mitochondrial oxidative stress in the hippocampus of CUMS mice.

### Mitochondrial oxidative stress might be involved in the downregulation of NLRP3 by catalpol

Current studies have reported that ROS derived from mitochondria is a key signal for regulating NLRP3 inflammasome activation^28^. To further investigate whether or not oxidative stress was involved in the regulation of NLRP3 by catalpol, mitochondrion-targeted antioxidant peptide SS31 was used during CUMS. After 5 weeks of CUMS, three components of NLRP3 inflammasome, including NLRP3, cleaved caspase-1, and ASC, were detected by western blot. As expected, NLRP3 and cleaved caspase-1 displayed a significant increase after CUMS compare to the control group (Fig. 4f, g), while the administration of SS31 reversed the upregulation of both remarkably (Fig. 4f, g). However, there was no significant difference in ASC between groups (Fig. 4h). The overexpression of mature IL-1β was also significantly downregulated by SS31 as shown in Fig. 4i. All these data hint that mitochondrial oxidative stress has a vital role in the activation of NLRP3 inflammasome. Thus, the downregulation of NLRP3 in CUMS mice by catalpol might be associated with mitochondrial oxidative stress and ROS.

### Mitochondrial oxidative stress might be involved in the downregulation of neuroinflammation by catalpol

**Fig. 5:**
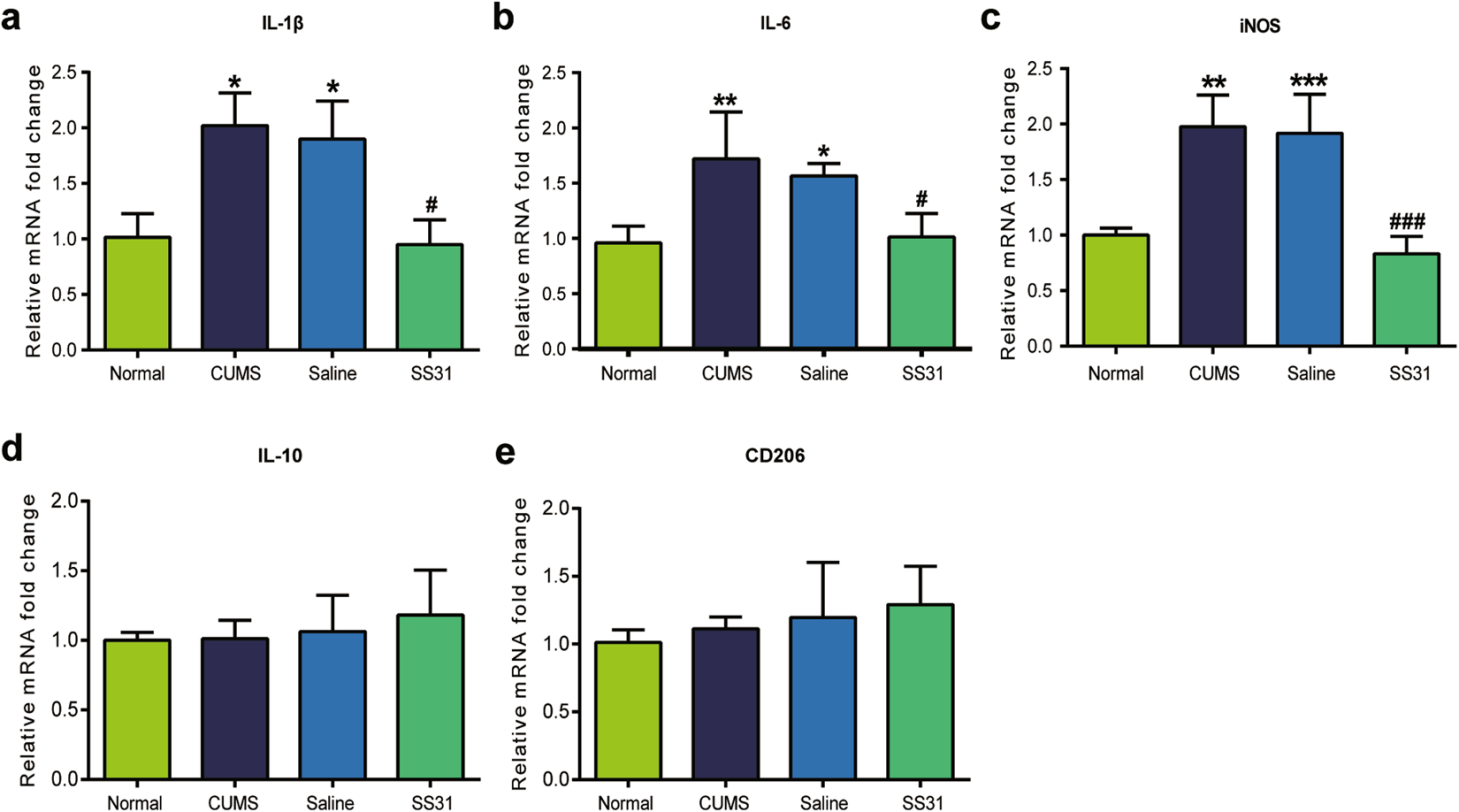
Mitochondrial oxidative stress might be involved in the downregulation of proinflammatory cytokines by catalpol. The expression of cytokines in hippocampus were assessed by qRT-PCR, the level of (**a**) IL-1β (*n* = 5–8/group), (**b**) IL-6 (*n* = 5–8/group), and (**c**) iNOS (*n* = 5–8/group) was downregulated significantly by catalpol. **d**, **e** There are no significant differences in the expression of (**d**) IL-10 (*n* = 5–8/group) and (**e**) CD206 (*n* = 5–8/group) between groups. All data are expressed as the mean ± SD. *p < 0.05, **p < 0.01, ***p < 0.001, compared with control group; ^#^*p* < 0.05, ^###^*p* < 0.001, compared with vehicle group.

The effect of SS31 on inflammatory cytokines was checked by examining the expression of IL-1β, IL-6, iNOS, IL-10, and CD206. As shown in Fig. 5, the expression of IL-1β, IL-6, and iNOS were significantly increased after the 5-week CUMS procedure (Fig. 5a–c). Our data revealed that SS31 intake significantly renormalized the overexpression of IL-1β, IL-6, and iNOS induced by CUMS (Fig. 5a–c). In addition, SS31 had no significant effect on the expression of either IL-10 or CD206 which are known as anti-inflammatory cytokines (Fig. 5d, e). We could infer from this observation that mitochondrial oxidative stress may play a key role in the regulation of CUMS-induced neuroinflammation by catalpol.

## Discussion

The CUMS procedure utilized in this study is widely employed for depression disorder research as it represents the pathophysiology of human depression^32–34^. CUMS murine model is characterized by deficits in behavioral tests, which includes a longer immobile time^35,36^. We confirmed that mice exposed to CUMS became more immobile and struggle less during the FST, exhibited a decline in rearing behaviors during OFT, and spent less time in the open arms during the EPM test which is consistent with our previous study^37^. In our present study, catalpol was found to effectively improve behavioral deficits in FST and EPM test as well as anxiety-like behaviors in OFT test without any impairment of locomotor activity. Our results indicated that catalpol may have antidepressant effects on CUMS mice.

The NLRP3 inflammasome complex is a molecular mechanism that translates psychological stressful stimuli into inflammatory responses. NLRP3 inflammasome-mediated inflammation is a familiar characteristic of various diseases in the CNS^38^. Recent researches have demonstrated that NLRP3 activation is the key factor in the pathogenesis of depression disorders^39–41^. As the most studied inflammasome, NLRP3 inflammasome contains an NLRP3 that recruits the ASC adapter protein through its PYD domain, followed by caspase-1 binding to the ASC through its CARD domain^42^. The assembly of NLRP3 complex is responsible for the proteolytic cleavage of pro-IL-1β into the active and secreted forms IL-1β, driving proinflammatory responses that culminate in cellular damage^43^. IL-1β might be the driving force in acute neuroinflammation, which marked the start of the proinflammatory responses targeted specifically at psychological stress by resulting in a cascade of inflammatory cytokine responses^44,45^. It has been speculated that depression is related to an increased secretion of cytokines, in particular IL-1β^46^. Therefore, the expression of NLRP3 inflammasome and IL-1β were evaluated. Increasing level of NLRP3 inflammasome were downregulated following the administration of catalpol, seen as the changes in NLRP3 and cleaved caspase-1. Overexpression of IL-1β induced by CUMS was significantly reversed by catalpol. Moreover, we used NLRP3 KO mice to determine the role NLRP3 played in depression. We found that *Nlrp3−/−* could partly inhibit the depressive- and anxiety-like behaviors induced by CUMS. Our results provided evidence that NLRP3 inflammasome is involved in the antidepressant effects of catalpol on CUMS mice.

Neuroinflammation, defined as the cellular and biochemical responses to numerous insults that occur within the CNS^12^, has been shown to be highly involved in depression^47^. In the CNS, NLRP3 was found in microglial^48^. Moreover, in a CUMS model of depression, only the microglia appeared to express NLRP3^49^. Microglial is activated in neuroinflammation with an ameboid feature, which is characterized by enlarged cell bodies and synaptic retraction, contributing to the development of depression and produce pro/anti-inflammatory cytokines such as IL-1β, TNF-α, and IL-6^50,51^. Recent studies have shown that these cytokines play an important role in depression disorders^39,52,53^. In addition, TNF-α and IL-1β can further induce the production of other cytokines, including IL-6^54^. Microglia can polarize into either the classically activated M1 (proinflammatory) phenotype, exacerbating neuronal damage, or the alternatively activated M2 (anti-inflammatory) phenotype, exerting neuroprotection and promoting neuronal recovery^55,56^. In this study, we explored the expression of microglial activation biomarker at the end of CUMS procedure. iNOS, the marker of M1 microglial which is proinflammatory phenotype, was significantly overexpressed in CUMS mice. Catalpol administration caused a remarkable increase of CD206, marker of M2 (anti-inflammatory) microglial. Thus, promoting microglial polarization to the M2 phenotype and inhibition of proinflammatory cytokine production could be a viable strategy for the treatment of neuroinflammation-associated depression. The current study demonstrated that catalpol possessed anti-inflammatory effect on CUMS mice and inhibited microglial polarization to the M1 phenotype.

Mitochondrial dysfunction may contribute to increased oxidative stress in MDD^57^. ROS, which is associated with mitochondrial dysfunction, may be involved in the mechanism of NLRP3 activation^58^. Meanwhile, microglial activation may increase its production of proinflammatory cytokines which promotes ROS and accelerates oxidative stress in turn. Increased ROS products can increase proinflammatory production and enhance microglial activation via stimulating NF-κB^59^, creating a pathological positive feedback loop in some psychiatric disorders^60^. As expected, the expression of ROS was remarkably upregulated after CUMS procedure, and that catalpol, which was previously reported to have antioxidative stress effects in depression^11^, reversed the overexpressed ROS in CUMS mice. In this present study, a mitochondrion-targeted antioxidant peptide SS31 was used to ascertain the role of oxidative stress in NLRP3 inflammasome and simultaneous neuroinflammation. Interestingly, after SS31 treatment, the expression of IL-1β, NLRP3, and cleaved caspase-1 was inhibited by SS31, while there was no change in ASC. When compared with CUMS mice, SS31 completely inhibited the increasing expression of IL-6 (proinflammatory cytokine) and iNOS.

In this research, we report that the underlying mechanism of the antidepressive effect of catalpol may be related to neuroinflammation and oxidative stress in mitochondria. However, we only use male C57BL/6 mice, which is a limitation of the present study since recent study indicated that the susceptibility to depression differs between males and females^61^. In addition, a few markers of the proinflammatory microglia were investigated here, further experiments which focus on the link between regulation of microglia function phenotype and NLRP3 inflammasome and the role catalpol plays in the regulation of microglia need to be considered, for instance by using Tmem119Cre and NLRP3^fl/fl^ mice.
